# Evaluation of the effect of percutaneous mitral balloon valvuloplasty on left ventricular systolic functions using strain and strain rate echocardiography

**DOI:** 10.3906/sag-1901-69

**Published:** 2020-06-23

**Authors:** Ertuğrul Emre GÜNTÜRK, Oğuzhan BARAN, Hasan AKKAYA, Özcan ÖRSÇELİK

**Affiliations:** 1 Department of Cardiology, Faculty of Medicine, Niğde Ömer Halisdemir University, Niğde Turkey; 2 Department of Cardiology, Sivas Numune Hospital, Sivas Turkey; 3 Department of Cardiology, Faculty of Medicine, Mersin University, Mersin Turkey

**Keywords:** Mitral stenosis, mitral valvuloplasty, echocardiography, doppler, strain–strain rate

## Abstract

**Background/aim:**

This study aimed to evaluate the effect of successful percutaneous mitral balloon valvuloplasty (PMBV) on left ventricular systolic functions using strain and strain rate echocardiography in moderate–severe mitral stenosis (MS) patients with normal left ventricular systolic function confirmed by conventional echocardiography.

**Materials and methods:**

Patients with moderate–severe MS who had undergone successful PMBV were included. Conventional echocardiographic parameters were evaluated before and after PMBV. Peak systolic strain and strain rate values of basal, mid, and apical segments of the left ventricular anterior, inferior, septum, and lateral walls were determined.

**Results:**

After PMBV, significant decreases were determined in the peak and mean gradients of the mitral valve and pulmonary artery pressure, but a significant increase in the mitral valve area. Significant increases (improvement) were determined in the peak systolic strain and strain rate in the basal, mid, and apical segments of the left ventricular septum, lateral, anterior, and inferior walls and in the left ventricular global peak systolic strain (–17.32 ± 0.58% vs. –12.38 ± 1.06%) and strain rate (–1.65 ± 0.07 vs. –1.22 ± 0.12).

**Conclusion:**

Strain and strain rate echocardiography can be used for quantitative evaluation of the effect of PMBV on the left ventricular systolic functions in moderate–severe MS patients.

## 1. Introduction

Mitral stenosis (MS) is a valvular heart disease that remains as an important cause of morbidity and mortality worldwide, particularly in developing countries. It generally appears as a late term complication of acute rheumatic fever [1]. Mitral stenosis leads to hemodynamic disorders by inhibiting left ventricular filling. Disease progression results in left ventricular dysfunction, pulmonary hypertension, and right heart failure [2]. In general, depending on the patient’s status, percutaneous intervention or surgery is recommended for the treatment of moderate–severe MS patients (mitral valve area <1.5 cm2) and/or symptomatic patients [3]. Percutaneous mitral balloon valvuloplasty (PMBV) is an effective therapeutic option for the treatment of MS and is successfully performed in selected MS patients based on hemodynamic and echocardiographic criteria [4,5].

Echocardiography is the method of choice for the diagnosis of MS and the assessment of disease severity [6]. The opportunity for more detailed and comprehensive examination has arisen with technological developments and the availability of 3-dimensional echocardiography [7]. Strain and strain rate echocardiography, a method based on the principal of tissue Doppler, has been defined for the quantification of regional myocardial function [8]. Strain and strain rate echocardiography came into use in the late 1990s as a method of measuring ventricular performance [9]. Strain defines the change in the size of an object, or deformation, as a result of force applied to that object, and is expressed as percentage (%); strain rate is the rate of deformation [9]. It is thought that strain and strain rate echocardiography may enhance the accuracy, quality, and reproducibility of test interpretations and diagnostic performance by allowing quantitative evaluation of ventricular wall movements [10]. The role of strain and strain rate echocardiography in clinical practice would be better understood with an increased number of comparative studies. The aim of this study was to investigate the effect of successful PMBV on left ventricular systolic functions by strain and strain rate echocardiographic parameters using tissue Doppler in moderate–severe MS patients with normal left ventricular systolic functions confirmed by conventional echocardiography techniques.

## 2. Materials and methods

This prospectively designed study included patients with moderate–severe MS who had undergone a successful PMBV procedure in our clinic within a 10–month period. Patients with diabetes mellitus, arterial hypertension, coronary artery disease, chronic renal failure, atrial fibrillation, left ventricular ejection fraction (EF) <50%, segmental wall movement abnormalities, left bundle branch block, moderate–severe aortic stenosis, aortic insufficiency, moderate–severe tricuspid stenosis, and those who developed mechanical complications such as severe mitral regurgitation, pericardial tamponade, and cardiac perforation during PMBV were excluded. The study was approved by the Ethics Committee of the Erciyes University School of Medicine and informed consent was obtained from all the patients.

Echocardiography was performed both before and one month after the PMBV. Transthoracic echocardiographic images of the patients were recorded at end–expiration by the same surgeon using the 2.5 MHz electronic transducer of a Vivid 7 Echocardiography device (GE Vingmed Ultrasound, Horten, Norway) while patients were lying in the left lateral recumbent position. The left atrium diameter and the left ventricular systolic and diastolic diameters were measured on the parasternal long axis images, and EF and fractional shortening (FS) were measured using the Teichholz method. Planimetric valve area was measured on the parasternal short axis images. EF was calculated using Simpson’s rule on the apical 4– and 2–chamber images; the peak and mean mitral valve gradient was calculated using continuous wave (CW) Doppler, and mitral valve area was calculated using the pressure half–time (PHT) method. In addition, pulmonary artery systolic pressure was measured using the CW Doppler tracing of the tricuspid regurgitation jet. The right ventricle size was also measured. The presence and degree of mitral insufficiency, aortic insufficiency, and tricuspid regurgitation were determined using a color tissue Doppler. Color tissue Doppler images were obtained from the apical 4– and 2–chamber views at a frame rate of >90 frame/s and recorded at the end–expiration over 3 respiratory cycles. The peak systolic strain and peak systolic strain rates of the basal, mid, and apical segments of the left ventricular anterior, inferior, septum, and lateral walls were obtained on the apical 4– and 2–chamber tissue Doppler images and left ventricular global peak systolic strain and strain rates were calculated with their mean values.

### 2.1. Statistical analysis

Data were analyzed using the Statistical Package for the Social Sciences version 15 for Windows software (SPSS Inc., Chicago, IL, USA). Conformity of the variables to normal distribution was assessed with the Shapiro–Wilks test. Because the data was distributed normally, statistical data were stated as mean ± standard deviation (X ± SD) and paired sample t test was used to compare the variables before and after PMBV. A P-value <0.05 was considered statistically significant.

## 3. Results

Evaluation was made of 30 patients, comprising 23 females and 7 males with a mean age of 46 ± 11 years. Conventional echocardiography findings of the patients before and after PMBV are presented in Table 1. There were significant decreases in the mitral valve peak gradient, mean gradient, and pulmonary artery pressure after PMBV compared with those values before PMBV. A significant increase was determined in the mitral valve area after PMBV, which was measured using planimetry and the PHT method. There was no significant change in the EF and FS after PMBV. Peak systolic strain values and strain rates before and after PMBV are presented in Table 2 and Table 3, respectively.

**Table 1:** Conventional echocardiography findings of patients before and after percutaneous balloon valvuloplasty.

	Before PMBV Mean ± SD	After PMBV Mean ± SD	P
LVDD, cm	4.66 ± 0.62	4.68 ± 0.46	0.88
LVSD, cm	3.19 ± 0.54	2.98 ± 0.42	<0.05
EF% (Teichholz method)	62.57 ± 7.60	65.46 ± 5.84	0.12
EF% (Simpson’s rule)	61.80 ± 5.29	62.03 ± 3.65	0.66
FS %	33.75 ± 6.09	37.59 ± 4.56	0.06
LA, cm	4.62 ± 0.74	4.34 ± 0.87	0.06
RV, cm	3.58 ± 0.40	3.51 ± 0.41	0.21
PAP, mmHg	48.83 ± 15.11	36.62 ± 8.93	<0.05
Peak Gradient, mmHg	20.36 ± 7.29	10.33 ± 2.01	<0.05
Mean Gradient, mmHg	11.61 ± 3.36	5.12 ± 1.28	<0.05
MVA, planimetry, cm2	1.16 ± 0.19	1.93 ± 0.28	<0.05
MVA, PHT, cm2	1.09 ± 0.21	1.82 ± 0.25	<0.05

PMBV: Percutaneous mitral balloon valvuloplasty; LVDD: Left ventricular diastolic diameter; LVSD: Left ventricular systolic diameter; EF: Ejection fraction; FS: Fractional shortening; LA: Left atrium; RV: Right ventricle; PAP: Pulmonary artery pressure; MVA: Mitral valve area; PHT: Pressure half-time; SD, Standard deviation.

**Table 2:** Strain values before and after percutaneous mitral balloon valvuloplasty.

	Peak systolic strain, %		P
	Before PMBV Mean ± SD	After PMBV Mean ± SD	
Myocardial wall segment			
Lateral			
Basal	–12.58 ± 1.59	–16.47 ± 3.10	<0.05
Mid	–12.51 ± 1.82	–16.91 ± 1.05	<0.05
Apical	–11.10 ± 1.50	–17.22 ± 0.97	<0.05
Septum			
Basal	–13.41 ± 1.96	–16.11 ± 0.96	<0.05
Mid	–12.21 ± 1.77	–16.79 ± 2.98	<0.05
Apical	–12.44 ± 1.72	–17.21 ± 1.01	<0.05
Anterior			
Basal	–13.68 ± 1.75	–18.11 ± 1.09	<0.05
Mid	–12.59 ± 1.52	–18.56 ± 1.07	<0.05
Apical	–12.54 ± 1.73	–17.34 ± 1.03	<0.05
Inferior			
Basal	–11.86 ± 1.33	–18.07 ± 0.91	<0.05
Mid	–10.90 ± 1.48	–17.44 ± 0.87	<0.05
Apical	–12.44 ± 1.99	–16.64 ± 1.01	<0.05
Left ventricular global	–12.38 ± 1.06	–17.32 ± 0.58	<0.05

PMBV: Percutaneous mitral balloon valvuloplasty; SD, Standard deviation.

**Table 3:** Strain rates before and after percutaneous mitral balloon valvuloplasty.

	Peak systolic strain rate, s-1		P
	Before PMBV Mean ± SD	After PMBV Mean ± SD	
Myocardial wall segment			
Lateral			
Basal	–1.05 ± 0.21	–1.48 ± 0.11	<0.05
Mid	–1.13 ± 0.13	–1.55 ± 0.12	<0.05
Apical	–1.18 ± 0.15	–1.64 ± 0.13	<0.05
Septum			
Basal	–1.24 ± 0.22	–1.55 ± 0.13	<0.05
Mid	–1.11 ± 0.14	–1.51 ± 0.19	<0.05
Apical	–1.21 ± 0.14	–1.62 ± 0.12	<0.05
Anterior			
Basal	–1.22 ± 0.17	–1.76 ± 0.16	<0.05
Mid	–1.24 ± 0.15	–1.67 ± 0.11	<0.05
Apical	–1.31 ± 0.11	-1.75 ± 0.12	<0.05
Inferior			
Basal	–1.18 ± 0.17	–1.53 ± 0.12	<0.05
Mid	–1.24 ± 0.21	–1.66 ± 0.08	<0.05
Apical	–1.25 ± 0.14	–1.76 ± 0.12	<0.05
Left ventricular global	–1.22 ± 0.12	–1.65 ± 0.07	<0.05

PMBV: Percutaneous mitral balloon valvuloplasty; SD, Standard deviation.

**Figure 1:**
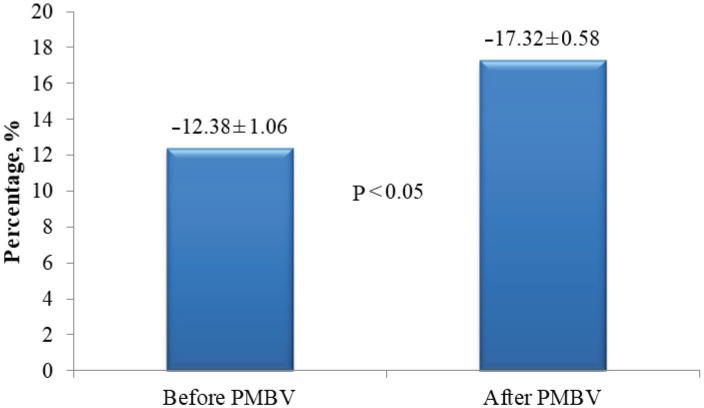
Changes in the left ventricular global peak systolic strain after percutaneous mitral balloon valvuloplasty.

**Figure 2:**
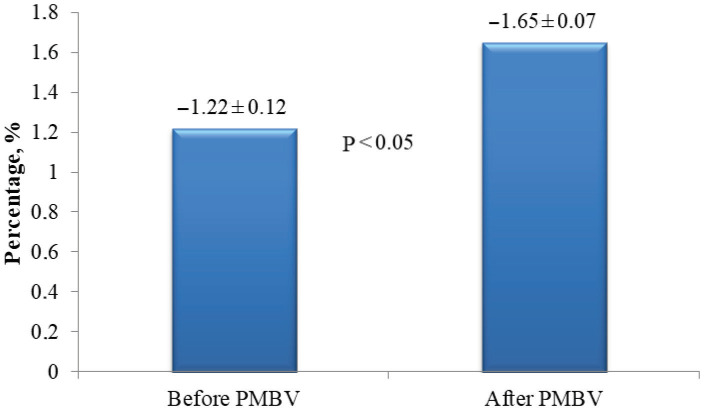
Changes in the left ventricular global peak systolic strain rate after percutaneous mitral balloon valvuloplasty.

A significant increase (improvement) was determined in the global peak systolic strain and strain rates of the septum, anterior, inferior, and lateral walls after PMBV. Changes in the left ventricular global peak systolic strain and strain rates are shown in Figure 1 and Figure 2, respectively.

## 4. Discussion

In the presence of mitral stenosis, left ventricular functions are influenced through numerous mechanisms and at various degrees. Although conventional echocardiography demonstrates that left ventricular functions are usually preserved in patients with pure MS, subclinical dysfunctions can be detected with the availability of new methods. Ozer et al. [11] conducted a tissue Doppler study in pure MS patients and a healthy control group and found the left ventricular global functions, to be similar in the 2 groups evaluated by FS, although systolic velocity, which was measured from the left ventricular septal and lateral mitral annulus, was found to be significantly lower in the MS group than in the control group. Similarly, Ozdemir et al. [12] conducted a tissue Doppler study and found the left ventricular wall annular velocity to be significantly lower in pure MS patients compared to healthy control subjects. In another tissue Doppler study by Sengupta et al. [13], it was determined that systolic and diastolic mitral annular velocities were lower in MS patients than in the healthy control group. It was also shown that these velocities increased after percutaneous mitral commissurotomy.

Strain and strain rate have been reported to be beneficial and sensitive parameters in assessing myocardial functions [14]. The fact that strain and strain rate measurements are not influenced by the tethering effect of adjacent myocardial segments is the most important advantage over tissue Doppler echocardiography. Strain and strain rate echocardiography has been clinically used in the management of cardiac resynchronization therapy, evaluation of systolic and diastolic functions, determination of myocardial ischemia and vitality, and identification of early-stage (subclinical) myocardial dysfunction and infarct segments [15–23]. The normal values of systolic longitudinal strain and peak systolic strain rate have been reported to be –19 ± 6% and –1.27 ± 0.39 s-1, respectively and the normal values of systolic radial strain and strain rate have been reported to be 41 ± 4.4% and 2.3 ± 0.3 s-1, respectively. Inter observer variability for strain and strain rate measurements has been found to be lower than 15% [24].

Subclinical left ventricular systolic dysfunction, which cannot be detected on M-Mod, 2–dimension echocardiography, or Doppler echocardiography, can be detected early using strain and strain rate echocardiography. Dogan et al. [25] conducted a tissue Doppler study on mild–moderate MS patients and a healthy control group and found peak systolic strain rate (1.2 ± 0.4 vs. 1.8 ± 0.39 s-1, P < 0.001) and end–systolic strain (10 ± 5 vs. 25 ± 6%) values to be significantly lower in MS patients compared to the control group (P < 0.001), although the MS patients had normal global systolic functions. In a study by Simsek et al. [26] conducted on pure MS patients with normal systolic functions and healthy control subjects, the values of systolic strain and strain rate in all segments of all myocardial walls were found to be significantly lower in the MS group. Bilen et al. [27] demonstrated deterioration in the left ventricular functions regardless of the hemodynamic severity of the stenosis in patients with MS compared to healthy individuals, using 2D strain and strain rate analyses.

The literature has a limited number of studies investigating the effect of PMBV on the left ventricular systolic functions using the strain and strain rate values. Dray et al. [28] demonstrated that strain and strain rate values obtained from lateral wall mitral annulus systolic velocity were significantly increased after PMBV in a 14–year–old girl with severe mitral stenosis, but normal left ventricular systolic functions were confirmed by conventional measurements. Based on that case, it was emphasized that strain and strain rate should be measured after surgery to demonstrate that left ventricular changes could be reversible in MS patients. Bektas et al. [29] measured the left ventricular long-axis strain and strain rate one day before and seven days after surgery in 30 patients with moderate–severe MS undergoing PMBV. Significant decreases were determined in the lateral, inferior, anterior, and septal systolic strain values after the surgery, although it was reported that there was no significant change in strain rate values. Sengupta et al. [30] compared preprocedure and postprocedure strain echocardiography findings of 57 patients with severe MS undergoing PMBV and determined a significant improvement in the global longitudinal strain and global circumferential strain values after PMBV. Barros–Gomes et al. [31] reported that global longitudinal strain values were strong predictors of long-term prognosis in MS patients with EF ≥ 50%, who underwent a successful PMBV.

The present study investigated the effect of PMBV on systolic strain and strain rate values in the basal, mid, and apical segments of the left ventricular lateral, septum, anterior, and inferior walls in adult patients with MS. Considering the conventional parameters, significant improvements were determined in the mitral valve area, mitral valve pressure gradient, and pulmonary artery pressure after PMBV. While there were no significant changes in EF and FS values, which are indicators of left ventricular systolic functions, significant increases (improvements) were determined after PMBV in systolic strain and strain rates in all segments of the myocardial walls in all patients. Thus, with the use of the strain and strain rate echocardiography technique, the present study quantitatively demonstrated that left ventricular systolic functions were improved after PMBV. The increase in left ventricular systolic strain and strain rate values might be related to the increase in preload and cardiac output, the decrease in afterload, the decrease in left ventricular wall stress, and the improvement in interventricular septum movement with decreasing pulmonary artery pressure.

Patients with mitral stenosis can remain asymptomatic for many years through self–limitation of effort capacity. However, the absence of symptoms does not change the fact that the disease is progressing. The symptoms may be subjective and misleading. In light of the present study, PMBV can be planned for patients who are asymptomatic except for having classical indications for PMBV but with subclinical left ventricular dysfunction detected using the strain and strain rate technique.

Limitations of the present study could be said to be the low number of patients and short follow-up period. In addition, the fact that tissue Doppler imaging is angle dependent can be considered another limitation.

In conclusion, left ventricular systolic dysfunction can be detected using the strain and strain rate technique in MS patients with normal left ventricular functions confirmed by conventional echocardiography techniques. Strain and strain rate echocardiography can be used as a quantitative method to assess the effect of PMBV on the left ventricular systolic functions in patients with moderate–severe MS.

## Acknowledgments

The study was approved by the Local Ethics Committee (Ethics Committee no: 2010/132) of Erciyes University.
